# Silencing subtelomeric *VSGs* by *Trypanosoma brucei* RAP1 at the insect stage involves chromatin structure changes

**DOI:** 10.1186/1756-8935-6-S1-P63

**Published:** 2013-03-18

**Authors:** Unnati M Pandya, Ranjodh Sandhu, Bibo Li

**Affiliations:** 1 Cleveland State University, Center for Gene Regulation in Health and Disease, Department of Biological, Geological, and Environmental Sciences, Cleveland, OH 44115, USA

## Background

*Trypanosoma brucei* causes human African trypanosomiasis and undergoes antigenic variation to evade its mammalian host immune attack. Throughout its life cycle, *T. brucei* is covered with glycoproteins on its cell surface. When infecting a mammalian host, bloodstream form (BF) *T. brucei* expresses bloodstream expression site (BES)-linked variant surface glycoproteins (VSGs) in a monoallelic fashion to ensure effectiveness of antigenic variation. In the midgut of its insect vector, tsetse, procyclic form (PF) *T. brucei* expresses procyclins. After migration to the tsetse’s salivary glands, *T. brucei* expresses metacyclic VSGs (mVSGs) to prepare for mammalian host infection. Regulation of *VSG* expression/silencing is critical for *T. brucei* virulence and its development. However the underlying mechanism of *VSG* regulation is not fully understood. Our lab identified a telomeric protein TbRAP1 as a key regulator of *BES-VSGs* in BF [1]. In this study we have further elucidated role of TbRAP1 in *VSG* regulation at PF stage and its possible underlying mechanism through chromatin remodeling.

## Materials and methods

We depleted TbRAP1 in PF cells using an inducible RNA interference (RNAi) approach. Quantitative RT-PCR was performed to analyze changes in steady state mRNA levels of BES-lined and metacyclic *VSGs* after depletion of TbRAP1. In order to test the effect of TbRAP1 depletion on chromatin structure, Formaldehyde-Assisted Isolation of Regulatory Elements (FAIRE) analysis and Micrococcal-nuclease digestion assays were performed.

## Results

In this study, we found that depletion of *Tb*RAP1 by RNAi led to derepression of *mVSGs* at both the BF and PF stages. Similar to that observed in BF cells, silencing of BES-linked *VSGs* in PF cells also depends on *Tb*RAP1. In addition, we found that silencing of BES-linked *VSGs* by *Tb*RAP1 is stronger in PF cells than that in BF cells. Furthermore, removal of *Tb*RAP1 led to a loosened chromatin structure at multiple loci in PF cells, particularly at the BES loci, but not in BF cells, indicating that different silencing mechanisms are used at different stages and that *Tb*RAP1-mediated silencing may involve modulation of chromatin structure in PF cells.

## Conclusions

Our observations confirm that *Tb*RAP1 is a key *VSG* silencer and that *Tb*RAP1 helps to determine the chromatin structure in multiple loci throughout the genome, particularly at BESs, at the insect stage.
